# RNA-seq analysis of bovine adipose tissue in heifers fed diets differing in energy and protein content

**DOI:** 10.1371/journal.pone.0201284

**Published:** 2018-09-20

**Authors:** Hilde K. L. Wærp, Sinéad M. Waters, Matthew S. McCabe, Paul Cormican, Ragnar Salte

**Affiliations:** 1 Department of Animal and Aquacultural Sciences, Norwegian University of Life Sciences, Ås, Norway; 2 Animal and Bioscience Research Department, Animal and Grassland Research and Innovation Centre, Teagasc, Dunsany, Co. Meath, Ireland; University of Illinois, UNITED STATES

## Abstract

Adipose tissue is no longer considered a mere energy reserve, but a metabolically and hormonally active organ strongly associated with the regulation of whole-body metabolism. Knowledge of adipose metabolic regulatory function is of great importance in cattle management, as it affects the efficiency and manner with which an animal converts feedstuff to milk, meat and fat. However, the molecular mechanisms regulating metabolism in bovine adipose tissue are still not fully elucidated. The emergence of next-generation sequencing technologies has facilitated the analysis of metabolic function and regulation at the global gene expression level. The aim of this study was to investigate the effect of diets differing in protein and energy density level on gene expression in adipose tissue of growing replacement dairy heifers using next-generation RNA sequencing (RNAseq). Norwegian Red heifers were fed either a high- or low-protein concentrate (HP/LP) and a high- or low-energy roughage (HE/LE) diet from 3 months of age until confirmed pregnancy to give four treatments (*viz*, HPHE, HPLE, LPHE, LPLE) with different growth profiles. Subcutaneous adipose tissue sampled at 12 months of age was analyzed for gene expression differences using RNAseq. The largest difference in gene expression was found between LPHE and LPLE heifers, for which 1092 genes were significantly differentially expressed, representing an up-regulation of mitochondrial function, lipid, carbohydrate and amino acid metabolism as well as changes in the antioxidant system in adipose tissue of LPHE heifers. Differences between HPHE and HPLE heifers were much smaller, and dominated by genes representing NAD biosynthesis, as was the significantly differentially expressed genes (DEG) common to both HE-LE contrasts. Differences between HP and LP groups within each energy treatment were minimal. This study emphasizes the importance of transcriptional regulation of adipose tissue energy metabolism, and identifies candidate genes for further studies on early-stage obesity and glucose load in dairy cattle.

## Introduction

The global human population is steadily increasing, and with it so are the demands for efficient animal production systems [1, 2]. Within the dairy industry, efficient meat and milk production is dependent on animals with a well-functioning metabolism and an efficient and purposeful nutrient allocation [3–5]. For growing cattle this implies a rapid growth rate without excess fattening, as the number of days spent to achieve productive size is proportional to total rearing costs [6], while excessive fattening has a negative impact on both feed efficiency, subsequent health and carcass value [5, 7]. High growth rates can be achieved by both breeding and feeding strategies. Growth is also affected by management and environmental conditions, although to a lesser degree [8, 9]. As the capacity for rapid genetic progress at a farm level is limited and management and environment yield smaller effects, nutritional management is the main strategy applied in practice. However, nutritionally induced high growth rates are associated with increased fatness [10]. There is a plethora of heifer studies reporting the effects of feeding regimes during the rearing period on growth rate and characteristics, as well as on subsequent milk yield in dairy heifers [11, 12]. However, the molecular mechanisms by which these effects are exerted are still not fully elucidated.

An important role of adipose tissue in this regard has emerged, as adipose is no longer considered merely a passive energy storage tissue, but is a transcriptionally active endocrine organ with an important role in whole-body metabolism and energy balance [13, 14]. When compared to monogastrics this is especially true for ruminants, in which adipose tissue is the main site for both lipid synthesis, storage and mobilization [15]. Increased knowledge of how transcriptomic regulation in adipose tissue is affected by nutrition in dairy heifers may contribute to the development of both feeding and breeding strategies to produce more efficient animals. Several studies on adipose tissue from cattle fed differently have been performed in the recent years, elucidating the connection between plane of nutrition and the expression of genes associated with lipogenesis, adipogenesis and lipolysis [16–18]. However, most gene expression studies on adipose tissue in cattle to date have been performed using microarrays and targeted gene analysis [14–16]. Recent increased availability and reduced costs of next generation sequencing techniques have led to more gene expression studies being performed by RNA-sequencing (RNAseq) technology. Compared to microarrays, RNA-seq is more sensitive and has a much wider dynamic range. It analyzes the whole transcriptome in an unbiased fashion, and is therefore not limited by predetermined target genes, array setups or a need for existing genome annotations [19].

Data in the published literature provides evidence of an effect of nutrition on adipose expression of genes, but until now such information have only been available through microarrays and targeted gene expression analyses performed on adult cows [16–18]. Hence, the objective of this study was to investigate the effects of diets differing in protein and energy density on the full adipose transcriptome of growing dairy heifers, and to identify gene networks related to critical metabolic pathways which can be targeted in further studies to enhance replacement heifer rearing strategies and production efficiency in the Norwegian Red cow.

## Materials and methods

### Ethics statement

All experimental procedures involving animals were approved by the Norwegian Animal Research Authority (FOTS ID 2955, Reference number: 2010/203231). Biopsies were harvested under epidural anesthesia, and all animals were given 3 mg/kg BW of Ketoprofen intramuscularly (Comforion vet., Orion Pharma Animal Health) to prevent pain or inflammation at the biopsy site.

### Animal model

This study was part of a large experiment designed to examine the effects of diets with differing energy and protein contents on growth characteristics, fat deposition and subsequent milk yield in Norwegian Red heifers, and from this to determine the optimal growth curve in the pre- and post-pubertal periods for a replacement dairy heifer. Eighty heifers from the dairy herd at the Norwegian University of Life Sciences (years 2010 and 2011) were assigned either to a high (HE) or low (LE) energy group, fed according to a BW gain of 800–1000 or 600–750 g/day from three months of age to confirmed pregnancy, respectively. Each energy group was further subdivided into two protein groups, i.e. low (LP) or high (HP) to give four dietary treatments with 20 animals in each group, *viz* low-protein high-energy (LPHE), high-protein high-energy (HPHE), low-protein low-energy (LPLE) and high-protein low-energy (HPLE). The university herd consists of two genetic groups, one group with high milk-yield (HMY) and one group with low occurrence of clinical mastitis (LCM) [20]. As this genetic difference could affect several variables of interest to our experiment, it was taken into account when allocating heifers to dietary treatments to generate groups which were balanced according to genetic group. Apart from this consideration, heifers were randomly allocated to treatment group. Body weights [21] were measured every second week, and body condition scores (BCS) and height at withers (WH) recorded every month for all experimental animals. The Norwegian 1–5 scale BCS system is based on Edmonson et al. [22] but calibrated for Norwegian Red [23].

#### Heifer feeding and management

All heifer calves were fed the same diet from birth to three mo of age. From three months of age until confirmed pregnancy, heifers were housed in a tie-stall barn and fed one of the four experimental diets. Fresh feed was offered twice daily and individual feed intake was recorded four days a week. HE groups were fed grass silage ad libitum whereas LE groups were fed the same silage mixed with 10 to 40% wheat straw (on a dry matter basis) given as restricted rations. All heifers were daily fed 1 kg of either of two custom-made concentrates, differing in protein content. Energy density of the diets was adjusted with the roughage quality. Diet protein content was adjusted with both roughage quality and type of concentrate. Table 1 gives the average nutrient content of roughage and custom made concentrates. Table 2 gives average daily intake of dry matter, energy, and protein for the different treatment groups.

**Table 1:** Average nutrient content of high- and low-energy (HE and LE) roughages and high and low protein (HP and LP) concentrates, all weighted by number of feed days during the experimental feeding period from 3 mo of age to confirmed pregnancy (g/kg of dry matter if not stated otherwise).

	Start experimental feeding to confirmed pregnancy			
	Roughage		Concentrate	
Variables^1^	HE (SD)	LE (SD)	LP	HP
Dry matter, g/kg	323 (40.8)	403 (48.9)	856	855
Ash	68.0 (8.0)	61.5 (6.5)	87.8	98.1
Crude protein	140 (13.7)	107 (8.9)	155	233
Crude fat	34.0 (2.9)	27.9 (1.6)	44	53
Starch	.	.	372	263
NDF	542 (18.5)	630.7 (25.8)	176	178
Sugar	38.3 (30.5)	28.8 (23.7)	.	.
AAT_N8_	64	62	83	98
PBV_N8_	42	15	43	103
NEL_N8_, MJ/kg DM	7.0	6.5	7.6	7.7

^1^ Neutral detergent fiber (NDF). Amino acids absorbed in the small intestine (AAT_N8_), protein balance in rumen (PBV_N8_), and net energy lactation (NEL_N8_), are all calculated values (TINE Optifôr, (Volden, 2011)) at 8 kg DMI; to calculate these variables a weighted (by feed days) average for the 2 roughages (HE, LE) and concentrates (LP, HP) were entered into TINE Optifôr as fictive feeds; note that standard values have no SD. Crude protein was analysed as Kjeldahl Nitrogen x 6.25.

**Table 2:** Calculated average daily intake in the ration of dry matter (DMI, kg DM/d), net energy growth (NEG, MJ/d), crude protein (CP_intake, g/d), and amino acids absorbed in the small intestine (AAT, g/d) in the 4 treatment groups (high-energy low-protein (LPHE), high-energy high-protein (HPHE), low-energy low-protein (LPLE), low-energy high-protein (HPLE) during the entire experimental feeding period.

Stage^1^	Treatment	Variables			
		DMI	NEG	CP_intake	AAT
3 mo of age to onset of puberty	LPHE	5.3	31.3	771	485
	HPHE	5.2	30.7	825	510
	LPLE	5.1	28.0	580	432
	HPLE	5.0	27.3	637	456
Puberty to confirmed pregnancy	LPHE	7.9	46.4	1,067	665
	HPHE	7.8	45.6	1,118	693
	LPLE	7.4	39.5	785	558
	HPLE	7.2	39.4	840	578

^1^Norwegian Red heifers reach puberty at about 280 kg regardless of age. HE animals entered puberty at 9 months of age, whereas LE animals entered puberty at 11.5 months of age. Pregnancy start was targeted at 400 kg BW for all heifers, and therefore confirmed pregnancy occurred later for LE heifers than for the faster-growing HE heifers (17 and 13 months of age, respectively).

### Adipose tissue biopsy sampling

Adipose tissue biopsies were collected at 12 months of age. The heifers were given a low epidural anesthesia of 3–5 ml of a local anesthetic (Lidokel-Adrenalin vet, Kela Laboratoria NV) prior to sampling. Subcutaneous adipose tissue biopsies were harvested lateral to the tail base. Each biopsy consisted of 0.5–1.5 g of adipose tissue. Biopsies were immediately snap frozen in liquid nitrogen, and subsequently stored at -80°C.

### RNA extraction and sequencing

Each adipose tissue biopsy was crushed on dry ice, and 100 mg was used for RNA extraction. RNA extraction was carried out using the Qiagen RNeasy lipid tissue mini kit (Qiagen). All samples were homogenized in Trizol using a PRO 200 handheld homogenizer (PRO Scientific Inc., Oxford, CT USA). Except for the homogenization step, the RNA extraction methodology was performed according to the manufacturer’s protocol. The quantity and quality of RNA was determined using a Nanodrop spectrophotometer ND-1000 (Nanodrop Technologies, Wilmington, DE, USA) and the RNA 6000 Nano Lab Chip kit (Agilent Technologies Ireland Ltd., Dublin, Ireland), respectively. The best-quality RNA samples to give a balanced sample set with respect to genetic group and dietary treatment were selected for further RNA sequencing (n = 6 for LPHE, HPHE, LPLE and HPLE, respectively). RIN values for these samples varied between 7.8 and 9.2. To avoid DNA contamination, all RNA samples were subjected to a DNAse treatment after extraction (Turbo DNA-free, Ambion, Life Technologies) and a subsequent clean-up (RNA Clean and Concentrator -25, Zymo Research Corp.).

Library preparation was performed using the Illumina TruSeq RNA sample prep kit v2, according to the manufacturer’s instructions (Illumina, San Diego, CA, USA). Sequencing was carried out on an Illumina HiSeq 2000 workstation, with 100 bp paired end reads, and the 48 samples distributed randomly over 12 lanes (Clinical Genomics, Toronto).

### Statistics and bioinformatics

The fastq files containing the raw sequence reads were examined using the software FASTQC (v 0.11.2.). Adapters were trimmed from all sample fastq files using Cutadapt (v 1.3). Reads were simultaneously quality trimmed by removing reads shorter than 20 bp, or with Phred scores below 25. Following adapter and quality trimming, all files were checked again in FASTQC. Paired-end read files were aligned to the UMD3.1 bovine genome assembly [24], using Tophat 2.0.12 [25]. The number of reads per gene in each sample was counted using HTSeq-count (v 0.6.1) [26]. Statistical analysis of read counts were carried out in EdgeR (v 3.1.2), a Bioconductor software package run in the statistical software environment R (v 3.1.2) [27]. Differential gene expression was tested in EdgeR using tagwise dispersions. The statistical tests were corrected for multiple testing using the Benjamini-Hochberg method as implemented in EdgeR. A generalized linear model which included dietary treatment and genetic group was fitted to the data using the glmFit function [28]. The comparisons were performed as pairwise comparisons and the differences between contrasting treatments, i.e. LPHE-LPLE, HPHE-HPLE, LPHE-HPHE and LPLE-HPLE, were tested. Significantly differentially expressed genes (DEG) were called at a FDR (false discovery rate adjusted p-value as given in EdgeR) of 0.1, and retained for further analysis. Subsequently, the resulting lists of DEG from the low and high-energy HP/LP contrasts and the low and high-protein HE/LE contrasts were compared to identify any DEG common to both comparisons within an energy or protein level. This suggests this gene was affected by energy or protein level regardless of the level of the other dietary variable. The DEG were subsequently analyzed using Ingenuity^®^ Pathway Analysis (IPA^®^, QIAGEN Redwood City, www.qiagen.com/ingenuity) to identify affected biological pathways and cellular functions represented by the DEG. An affected pathway is defined as a pathway containing more DEG than expected by chance, given the number of genes belonging to the pathway. Significance level for the pathway analyses was set at p < 0.05. Pathways or functions specifically pertaining to irrelevant diseases, species or tissues were omitted.

## Results and discussion

### Animal performance

At 12 months, BW of heifers were significantly different (p < 0.05) between all dietary treatments, but less so between different protein treatments within an energy treatment than across energy treatments. Body condition scores were different between different energy treatments (p < 0.05), but not between protein treatments within an energy treatment. Height at withers were significantly different (p < 0.05) between all treatment groups, but the largest difference was found between different energy levels. Least-squares means of BW, BCS and WH at 12 months of age are shown in Table 3.

**Table 3:** LSMEANS of body weight(BW), body condition score (BCS) and withers height (WH) at 12 months for different treatment groups (SE).

	BW, kg	BCS, points	WH, cm
LPHE	363 (0.9)	3.99 (0.016)	114.3 (0.2)
HPHE	371 (1.1)	3.95 (0.017)	115.3 (0.2)
LPLE	295 (0.8)	3.63 (0.014)	111.7 (0.2)
HPLE	311 (0.8)	3.63 (0.013)	112.4 (0.2)

### Differential gene expression

**Fig 1:**
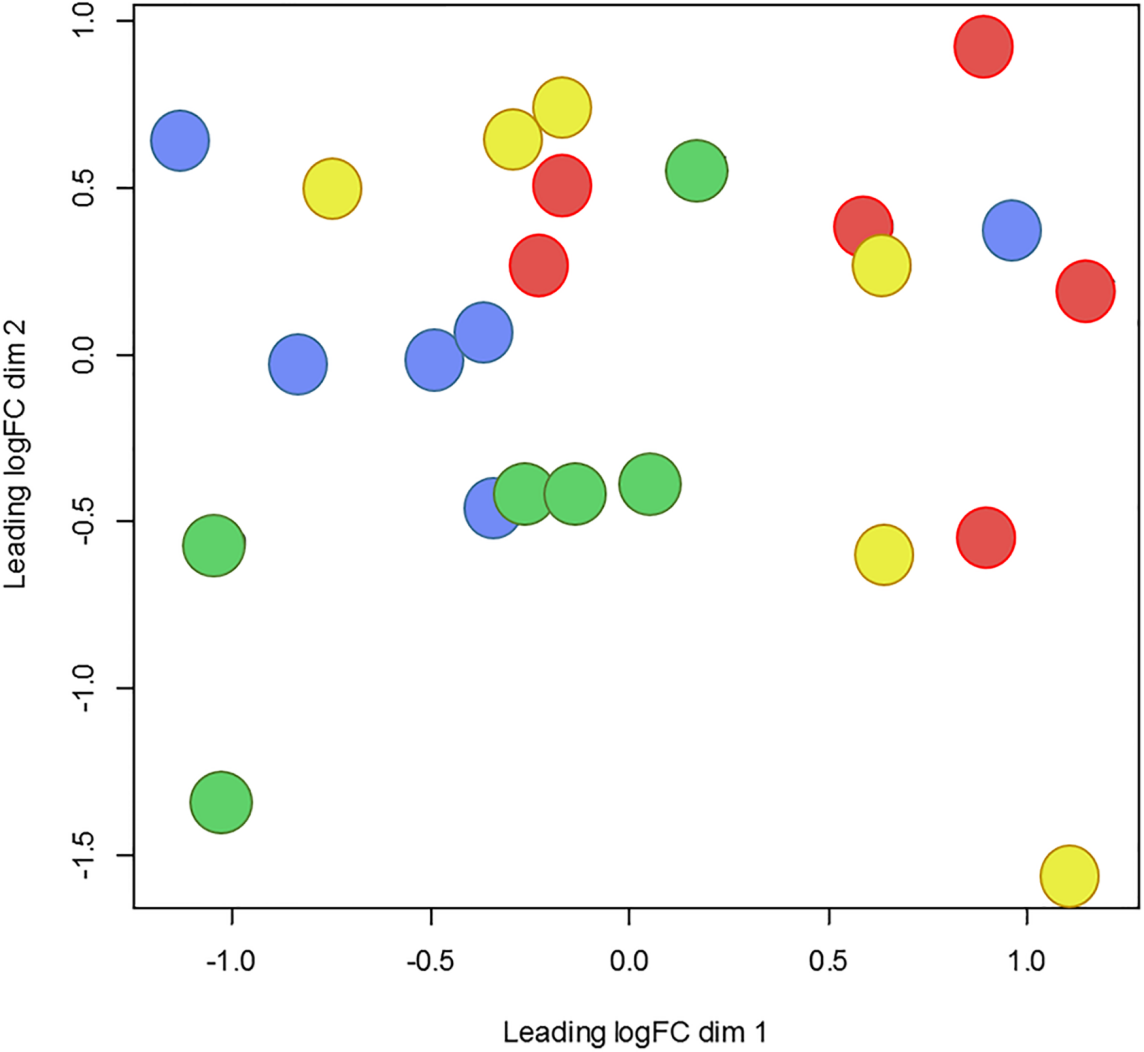
MDS plot of individual samples plotted 2-dimensionally by log fold change (logFC). Green = Low-protein, high-energy (LPHE), Blue = High-protein, high-energy (HPHE), Red = Low-protein, low-energy (LPLE), Yellow = High-protein, low-energy (HPLE).

Sequencing yielded on average 57.2 million raw reads per sample. Alignment analysis resulted in an average overall read mapping rate of 95.1% and a concordant pair alignment rate of 89.6%. Genes with a minimum CPM (counts per million) of two in at least three samples were considered to be expressed and were included in the differential expression analysis. Out of 26,740 gene transcripts in the UMD3.1 bovine genome assembly, 12,476 gene transcripts fulfilled this criterion. A multidimensional scaling plot visualizing the level of similarity between dietary treatments is presented in Fig 1. The greatest difference in adipose gene expression profiles was found between LPHE and LPLE heifers. This contrast yielded 1092 DEG, 40 times more than any other comparison. The number of DEG identified by contrasting treatments, energy or protein levels are shown in Table 4. Contrasts between the HP and LP groups within each energy level yielded only two DEG for the HE contrast, and one DEG for the LE contrast. Details on these DEG are presented in Table 5. The full lists of DEG between the LPHE-LPLE and the HPHE-HPLE contrasts are shown in S1 and S2 Tables, respectively. The RNAseq raw data are deposited and accessible through NCBI’s Gene Expression Omnibus with the GEO Series accession number GSE79347 (https://www.ncbi.nlm.nih.gov/geo/query/acc.cgi?acc=GSE79347).

**Table 4:** Number of up- and down-regulated differentially expressed genes between compared treatment groups.

Comparison	DEG total	Upregulated	Downregulated
LPHE-LPLE	1092	712	380
HPHE-HPLE	24	13	11
HPHE-LPHE	2	1	1
HPLE-LPLE	1	0	1
(LPHE-LPLE)–(HPHE-HPLE)	16	12	4
(LPHE-HPHE)—(LPLE-HPLE)	0	-	-

**Table 5:** DEG from HP-LP comparisons.

Gene symbol	Ensembl Gene ID	Description	logFC^a^	FDR^b^
HPLE-LPLE				
*CRYM*	ENSBTAG00000009842	crystallin, mu	-3.217	0.097394
HPHE-LPHE				
*ACTA1*	ENSBTAG00000046332	Actin, alpha 1, skeletal muscle	7.751	0.020466
*DSP*	ENSBTAG00000015106	Desmoplakin	-3.720	0.020466

^a^ logFC = log2 fold change

^b^ FDR = False discovery rate.

### Pathway analysis

**Fig 2:**
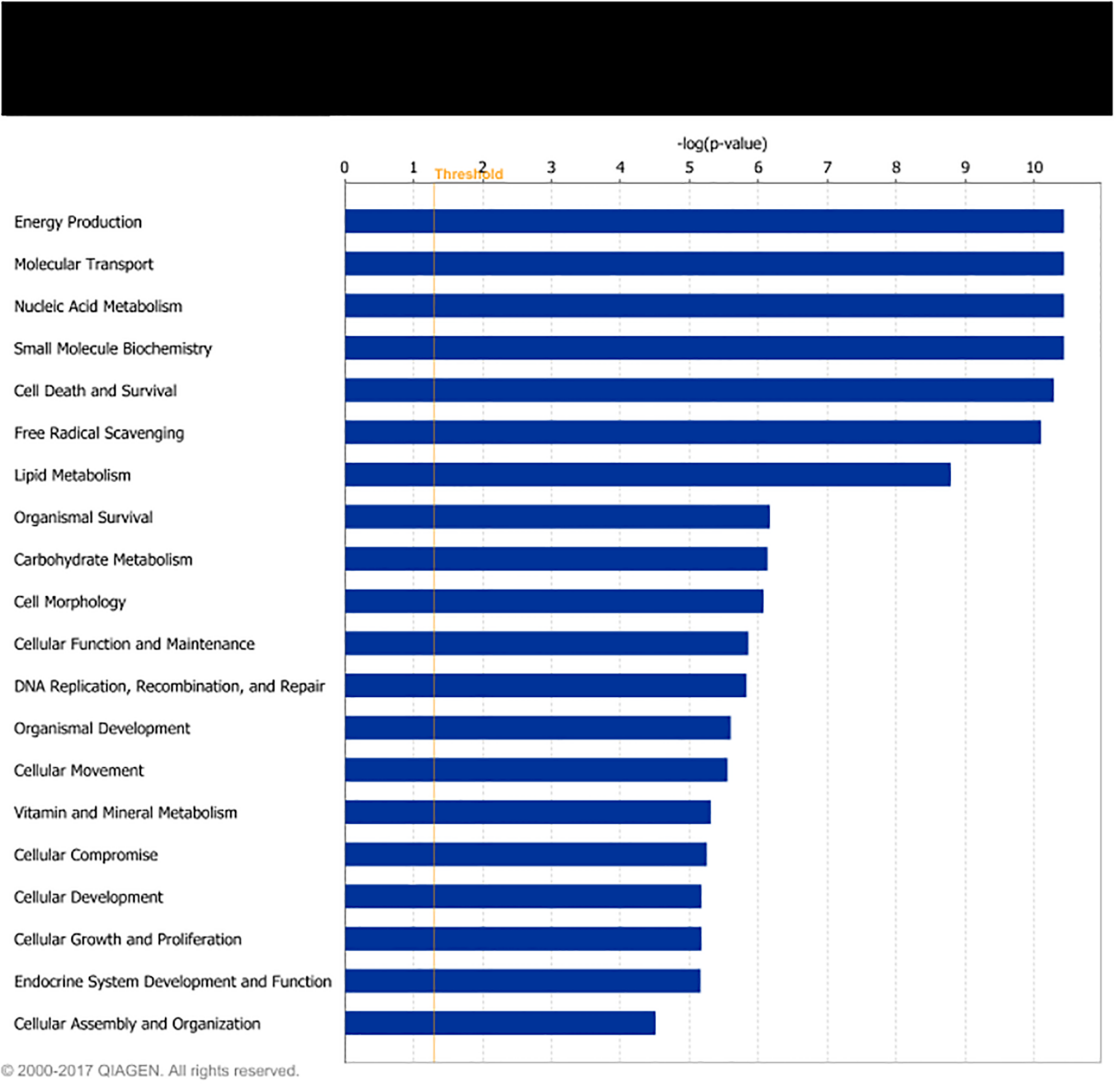
Cellular functions of differentially expressed genes between low-protein fed heifers on high- or low-energy diets. The bars indicate the likelihood [-log (p-value)] that the specific cellular function was affected by dietary energy level compared with other functions represented by the list of differentially expressed genes. Threshold–log p-value (orange line) is set to 1.3, which equals a p-value of 0.05.

**Fig 3:**
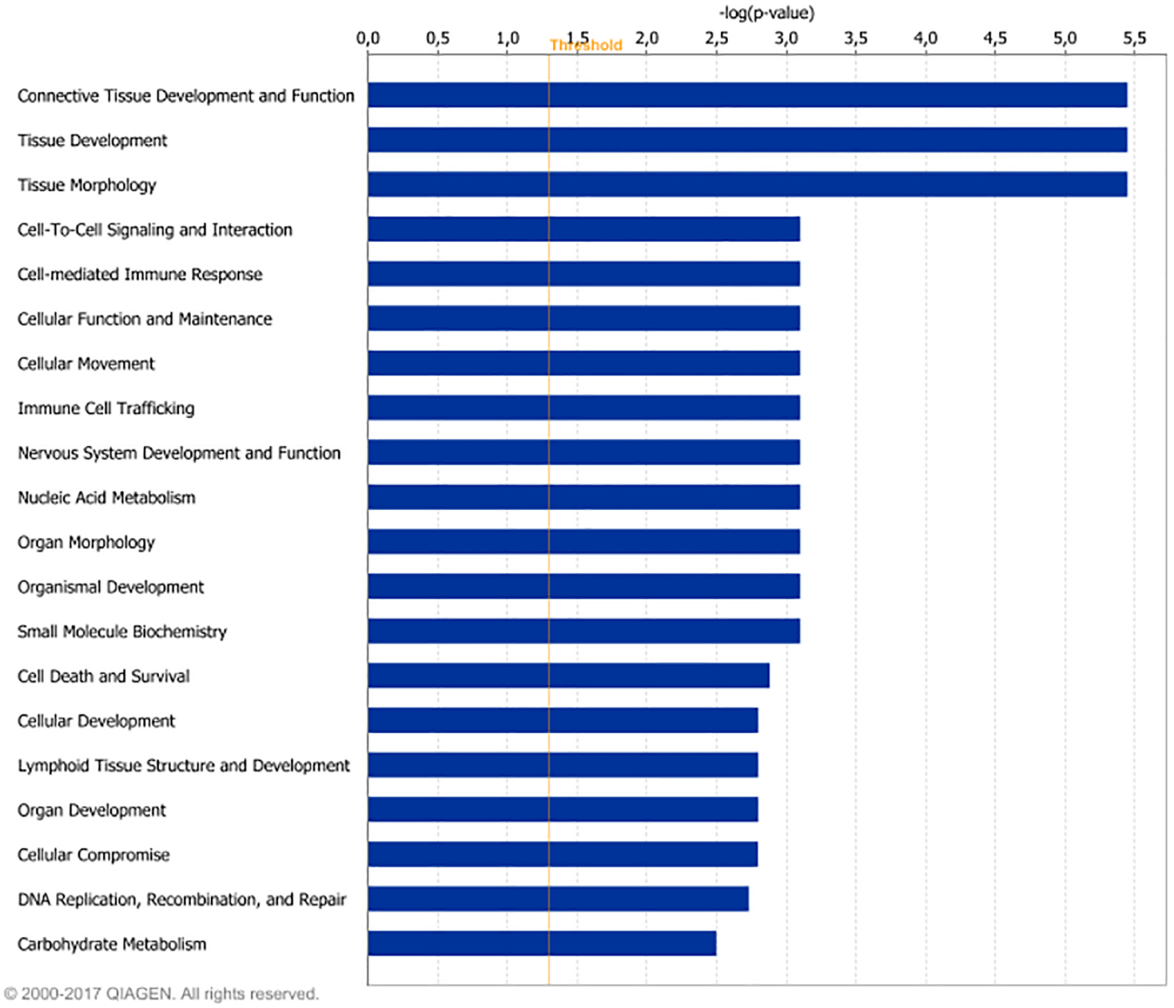
Cellular functions of differentially expressed genes between high-protein fed heifers on high-or low-energy diets. The bars indicate the likelihood [-log (p-value)] that the specific cellular function was affected by dietary energy level compared with other functions represented by the list of differentially expressed genes. Threshold–log p-value (orange line) is set to 1.3, which equals a p-value of 0.05.

**Fig 4:**
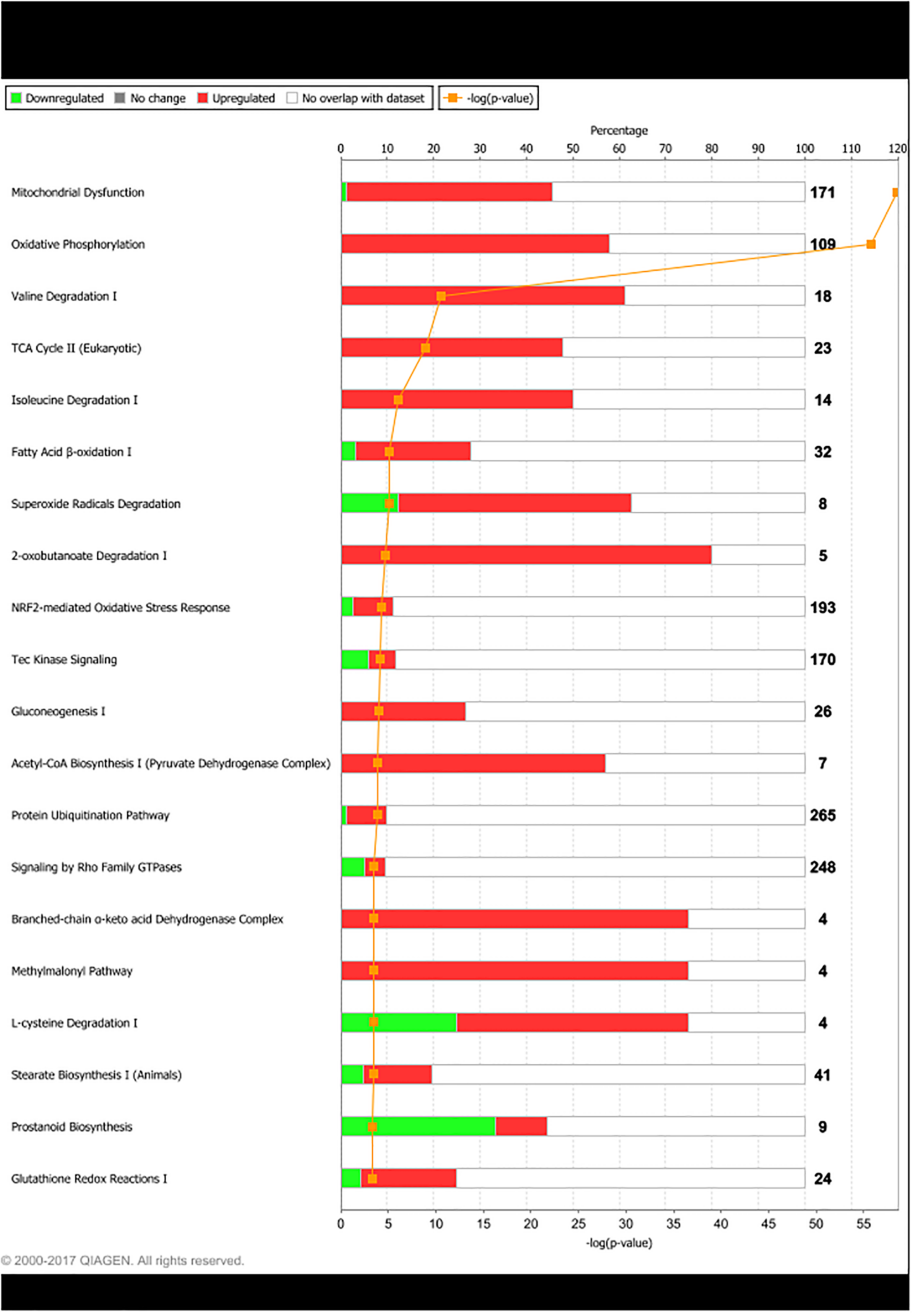
Main pathways differentially expressed between low-protein/ high-energy (LPHE) and low-protein/ low-energy (LPLE) fed heifers. Red bars indicate percent upregulated, and green bars indicate percent downregulated genes in the pathway in LPHE versus LPLE heifers. Number to the right of bars display total number of genes pertaining to each pathway. Orange squares indicate the negative logarithm of p-value of observation (-log p-value = 1.3 equals p-value = 0.05).

**Fig 5:**
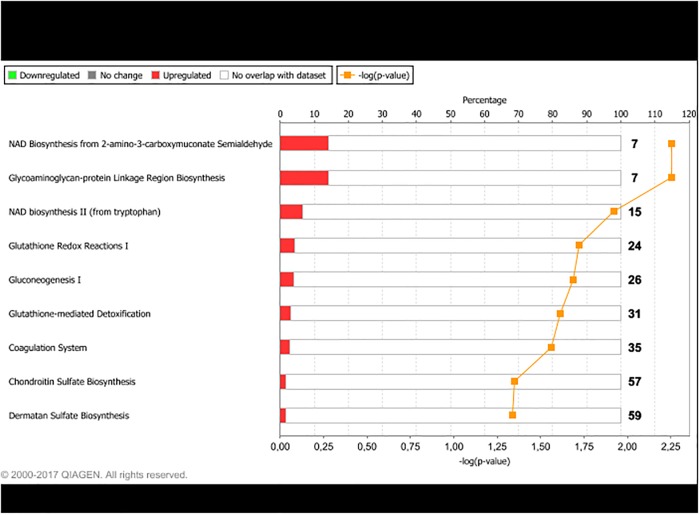
Main pathways differentially expressed between high-protein/ high-energy (HPHE) and high-protein/ low-energy (HPLE) fed heifers. Red bars indicate percent upregulated, and green bars indicate percent downregulated genes in the pathway in HPHE versus HPLE heifers. Number to the right of bars display total number of genes pertaining to each pathway. Orange squares indicate the negative logarithm of p-value of observation (-log p-value = 1.3 equals p-value = 0.05).

Differentially expressed genes were grouped in IPA according to their biological functions. The functional groups most highly represented by DEG found for LPHE-LPLE contrasts were nucleic acid metabolism, amino acid metabolism and small molecule biochemistry. Small molecule biochemistry was also the functional group most highly represented by the HPHE-HPLE DEG. Figs 2 and 3 display further cellular function differences found for DEG from the two LPHE-LPLE and HPHE-HPLE contrasts, respectively. Pathway analysis of the DEG between LPHE and LPLE heifers resulted in 99 significantly affected pathways (p < 0.05). The main pathways affected by the difference in LPHE and LPLE feeding regimes pertained to mitochondrial functions (mitochondrial dysfunction, oxidative phosphorylation and TCA cycle), amino acids (valine, isoleucine, leucine, tryptophan, arginine, proline and cysteine metabolism and degradation) and carbohydrate metabolism (pyruvate, propanoate, butanoate and ketone body metabolism, glycolysis and gluconeogenesis), antioxidant defense systems and fatty acid biosynthesis and degradation. The most highly affected pathways for this contrast are shown in Fig 4. Pathway analysis of the 24 DEG from the HPHE-HPLE comparison yielded the IPA pathways shown in Fig 5. Significantly affected pathways for this contrast were mainly related to glycosaminoglycan and NAD biosynthesis. NAD biosynthesis was also the main pathway represented by the 17 DEG common to both HE-LE contrasts. DEG from the two HP-LP contrasts were not subjected to pathway analysis due to low numbers.

### Gene expression differences between energy treatments

The massive difference in DEG numbers identified for the examined contrasts suggests a profound interaction between energy and protein level in the diet on adipose tissue metabolism, and that this interaction effect is in fact larger than the effects of protein or energy independently. The differences in gene expression between LPHE and LPLE heifers illustrates the importance of transcriptional regulation in metabolism, as several of the main affected pathways such as oxidative phosphorylation, TCA cycle, amino acid and carbohydrate metabolism clearly reflect changes in energy metabolism which are expected to differ in animals fed high- or low-energy diets. The DEG between HPHE and HPLE heifers suggest the main differences between these two groups was related to connective tissue structure (upregulated glycosaminoglycan synthesis by xylosyltransferase 1, *XYLT1*), and NAD synthesis by quinolinate phosphoribosyltransferase (*QPRT*). The low number of DEG in this contrast also affects the number of DEG common to both HE-LE contrasts, and leaves NAD synthesis as the pathway mainly affected by energy level regardless of protein level in the diet. This is an interesting finding, although not surprising, as nicotinamide adenine dinucleotide (NAD) is a coenzyme well known to take part in a plethora of cellular oxidation-reduction reactions, which constitute the basis of cellular energetics [29]. Based on the results of other gene expression studies, we expected certain genes to be differentially expressed in both energy contrasts. These genes included *FASN* [18, 30], *SCD* [18, 30], *ELOVL6* [30], *ACACA* [18, 30] and *OB* [31]. We would like to emphasize that even though these “classical” and expected DEG from the LPHE-LPLE contrast, did not obtain an FDR < 0.1 in the HPHE-HPLE contrast, 991 of the 1092 DEG from the LP comparison displayed the same directional change in the HP contrast. Thus, differences were mainly the same, only smaller, and given a bigger sample set, the results for the two contrasts would probably be more similar. For comparative purposes, we also performed an overall comparison of all HE animals against all LE animals within the same fitted model as described above. With an FDR cutoff value of 0.05 (because of the increased power of this test), the resulting DEG list was strikingly similar to the DEG list for the LPHE-LPLE contrast. In the following 4 sub-sections the main differences in adipose gene expression between LPHE and LPLE heifers are discussed.

#### Mitochondrial function

**Fig 6:**
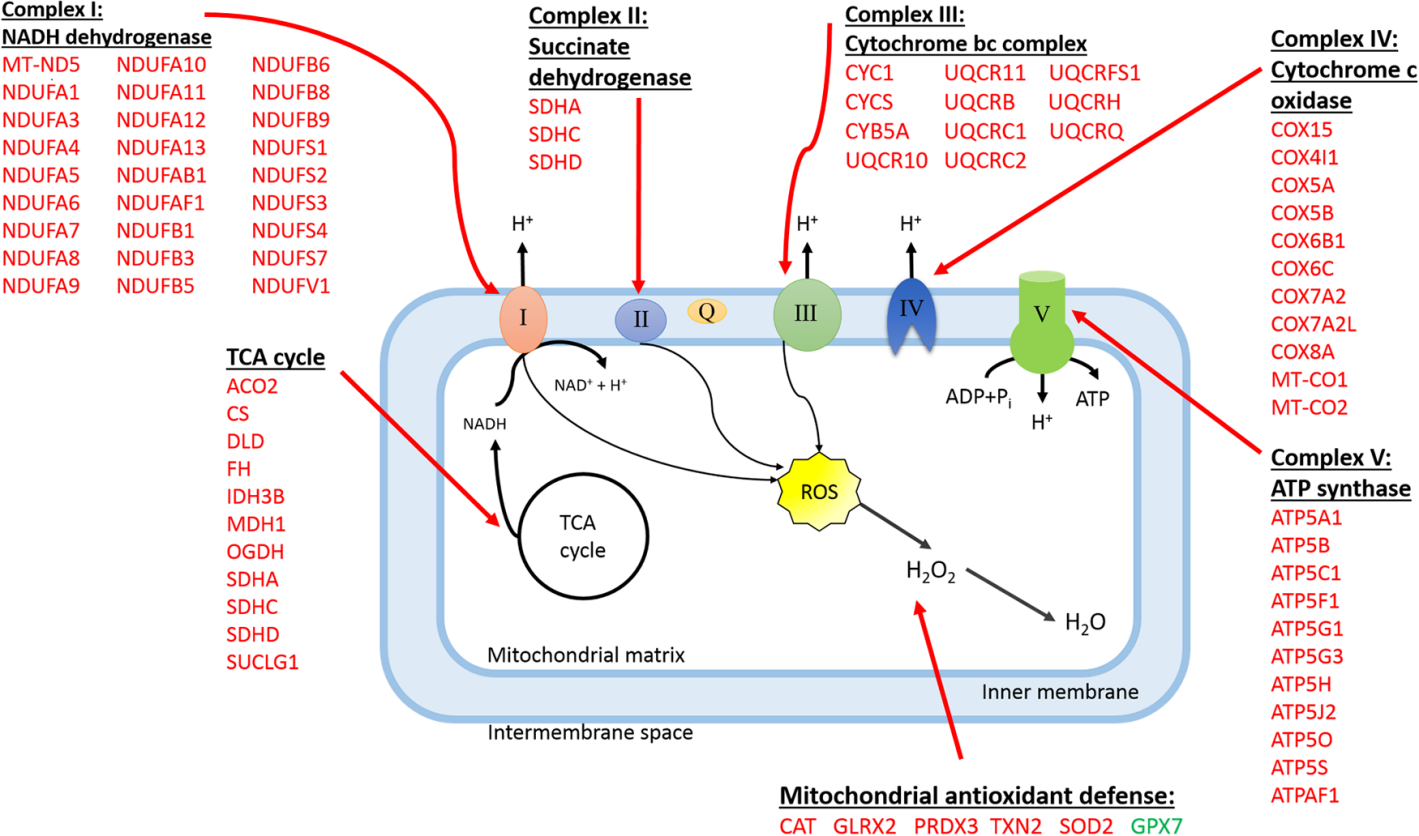
Genes associated with mitochondrial function, differentially expressed between low-protein fed heifers on high- (LPHE) or low- (LPLE) energy diets. Red colour indicate upregulated, and green colour indicate downregulated genes in LPHE versus LPLE heifers.

The tricarboxylic acid (TCA) cycle and the electron transport chain performing oxidative phosphorylation take place in the mitochondria. The TCA cycle is central to many anabolic and catabolic pathways in a mammalian cell as its main substrate, acetyl-CoA is produced from the initial breakdown of carbohydrates, protein and lipids. It also provides substrates for the synthesis of fatty acids, sterols, amino acids and glucose. The cycle consists of eight steps. Genes associated with all eight steps were up-regulated in LPHE heifers which indicates increased mitochondrial citrate production in LPHE heifers. Citrate is the main fatty acid precursor originating from oxidative metabolism. Citrate destined for fatty acid synthesis is transported out of the mitochondria by a citrate transport protein encoded by *SLC25A1*, which was up-regulated in LPHE heifers. In the cytosol, citrate is converted to acetyl-CoA by ATP citrate lyase (*ACLY*). In ruminants, acetate (from ruminal fermentation) has been shown to be the main carbon source for fatty acid synthesis [32]. Citrate lyase has traditionally been regarded as virtually inactive in ruminant adipose tissue, because of the low levels of glucose available for metabolism in peripheral tissues [32]. However, *ACLY* was up-regulated in LPHE compared to LPLE heifers. Recent findings, including this study show that the *ACLY* gene displays expression differences in the adipose tissue of cattle with different energy states [18, 30]. This suggests that citrate lyase does indeed play a part in ruminant metabolism and metabolic regulation, and may indicate an increased incorporation of glucose into fatty acids in animals with a positive energy balance. Genes associated with all of the 5 complexes in the electron transport chain were up-regulated in the LPHE heifers, indicating an increase in electron transport chain activity and energy (ATP) production. This makes sense as the role of adipose tissue as a metabolic excess energy endpoint requires it to deal with all forms of energy-yielding nutrients, transforming them in to lipids or to the energy required to produce these lipids. The genes associated with changes in mitochondrial function between LPHE and LPLE heifers, are presented in Fig 6.

#### Fatty acid biosynthesis and degradation

Differentially expressed genes associated with fatty acid synthesis and degradation are shown in Table 6. The up-regulation of genes associated with fatty acid synthesis (*ACACA*, *SCD*, *FASN* and *ELOVL6)*, fatty acid activation (*ACSL4*, *SLC27A3*, *SLC27A6*), and triacylglycerol (**TAG**) synthesis (*AGPAT2*, *DGAT2*, *ELOVL6*, *LPCAT4*, *PLPP3*) in the LPHE heifers is a reflection of their increased energy intake, leading to a higher production of TAG from citrate and other precursors than in the LPLE heifers. The activity of delta-9-desaturase is important in ruminant adipose tissue compared to monogastrics. This is because of the extensive microbial biohydrogenation of dietary unsaturated fatty acids occurring in the rumen, leaving almost exclusively saturated fatty acids (**SFA**) to be transported from viscera to the peripheral tissues in ruminants [33]. However, the high melting point of SFA makes them unsuitable as the sole source of fatty acids incorporated into cell membranes and adipose tissue. The high delta-9-desaturase activity of ruminant adipose tissue alleviates this effect, and makes oleic acid the most abundant FA in ruminant tissues [34].

**Table 6:** Fatty acid synthesis and degradation-associated genes differentially expressed between low-protein, high-energy heifers and low-protein, low-energy heifers.

*Symbol*	Ensembl	Entrez Gene Name	LogFC	FDR
*ACAA2*	ENSBTAG00000002863	acetyl-CoA acyltransferase 2	0,746	1,37E-02
*ACAT1*	ENSBTAG00000012885	acetyl-CoA acetyltransferase 1	0,866	1,94E-03
*ACSL4*	ENSBTAG00000018986	acyl-CoA synthetase long-chain family member 4	0,834	6,48E-02
*AGPAT2*	ENSBTAG00000025161	1-acylglycerol-3-phosphate O-acyltransferase 2	0,950	1,17E-02
*ALDH6A1*	ENSBTAG00000018469	aldehyde dehydrogenase 6 family, member A1	0,631	5,74E-02
*CYB5A*	ENSBTAG00000012012	cytochrome b5 type A (microsomal)	0,770	4,45E-02
*DGAT2*	ENSBTAG00000001154	diacylglycerol O-acyltransferase 2	0,628	6,36E-02
*DHCR24*	ENSBTAG00000004688	24-dehydrocholesterol reductase	0,544	4,32E-02
*DLAT*	ENSBTAG00000010709	dihydrolipoamide S-acetyltransferase	0,860	3,71E-03
*DLD*	ENSBTAG00000001908	dihydrolipoamide dehydrogenase	0,734	8,62E-03
*ECHS1*	ENSBTAG00000017710	enoyl CoA hydratase, short chain, 1, mitochondrial	0,943	1,95E-03
*EHHADH*	ENSBTAG00000019625	enoyl-CoA, hydratase/3-hydroxyacyl CoA dehydrogenase	0,730	4,70E-02
*ELOVL6*	ENSBTAG00000010564	ELOVL fatty acid elongase 6	1,586	2,44E-07
*FADS2*	ENSBTAG00000015505	fatty acid desaturase 2	-1,129	1,06E-03
*FASN*	ENSBTAG00000015980	fatty acid synthase	0,759	1,59E-02
*HSD17B10*	ENSBTAG00000017779	hydroxysteroid (17-beta) dehydrogenase 10	0,734	2,39E-03
*IDI1*	ENSBTAG00000004075	isopentenyl-diphosphate delta isomerase 1	1,316	2,52E-04
*IVD*	ENSBTAG00000004409	isovaleryl-CoA dehydrogenase	0,574	3,25E-02
*LPCAT4*	ENSBTAG00000020040	lysophosphatidylcholine acyltransferase 4	-0,597	8,63E-02
*LSS*	ENSBTAG00000018936	lanosterol synthase (2,3-oxidosqualene-lanosterol cyclase)	1,044	1,58E-03
*MVD*	ENSBTAG00000012059	mevalonate (diphospho) decarboxylase	0,785	5,88E-02
*NSDHL*	ENSBTAG00000009231	NAD(P) dependent steroid dehydrogenase-like	0,791	8,64E-04
*PDHA1*	ENSBTAG00000019852	pyruvate dehydrogenase (lipoamide) alpha 1	0,658	6,69E-03
*PDHB*	ENSBTAG00000021724	pyruvate dehydrogenase (lipoamide) beta	0,434	6,49E-02
*PLPP3*	ENSBTAG00000011640	phospholipid phosphatase 3	-0,820	1,84E-02
*SCD*	ENSBTAG00000045728	stearoyl-CoA desaturase (delta-9-desaturase)	1,102	3,90E-03
*SCP2*	ENSBTAG00000003746	sterol carrier protein 2	0,689	1,16E-02
*SLC27A3*	ENSBTAG00000021862	solute carrier family 27 (fatty acid transporter), member 3	-1,150	4,79E-03
*SLC27A6*	ENSBTAG00000004860	solute carrier family 27 (fatty acid transporter), member 6	0,935	8,21E-02
*THEM4*	ENSBTAG00000004772	thioesterase superfamily member 4	0,671	5,91E-02

#### Amino acid metabolism

Differentially expressed genes pertaining to amino acid metabolism are shown in Table 7. Genes associated with the metabolism and degradation of several amino acids (isoleucine, leucine, phenylalanine, valine, cysteine, lysine, alanine, tryptophan and aspartate) were up-regulated in the LPHE heifers. This is likely a general response, which would be present also in e.g. muscle because HE animals grow much faster than the LE heifers and thus have both an increasingly higher daily crude protein intake as well as an increased metabolism and degradation of amino acids.

**Table 7:** Amino acid metabolism-associated genes differentially expressed between low-protein, high-energy heifers and low-protein, low-energy heifers.

Gene symbol	Ensembl gene ID	Description	logFC	FDR
*AADAT*	ENSBTAG00000010326	aminoadipate aminotransferase	0,988	4,93E-04
*ABAT*	ENSBTAG00000004038	4-aminobutyrate aminotransferase	0,769	2,14E-02
*ACADSB*	ENSBTAG00000018041	acyl-CoA dehydrogenase, short/branched chain	0,749	7,26E-02
*ACAT1*	ENSBTAG00000012885	acetyl-CoA acetyltransferase 1	0,866	1,94E-03
*ALDH6A1*	ENSBTAG00000018469	aldehyde dehydrogenase 6 family, member A1	0,631	5,74E-02
*ALDH7A1*	ENSBTAG00000009646	aldehyde dehydrogenase 7 family, member A1	0,503	2,24E-02
*ASNS*	ENSBTAG00000003222	Asparagine synthetase	0,416	6.64E-02
*BCAT2*	ENSBTAG00000009172	branched chain amino-acid transaminase 2, mitochondrial	0,595	4,89E-02
*BCKDHA*	ENSBTAG00000016037	branched chain keto acid dehydrogenase E1, alpha polypeptide	0,669	1,44E-02
*BCKDHB*	ENSBTAG00000012096	branched chain keto acid dehydrogenase E1, beta polypeptide	0,737	1,47E-03
*CDO1*	ENSBTAG00000017442	cysteine dioxygenase type 1	-1,363	2,25E-02
*DLD*	ENSBTAG00000001908	dihydrolipoamide dehydrogenase	0,734	8,62E-03
*ECHS1*	ENSBTAG00000017710	enoyl CoA hydratase, short chain, 1, mitochondrial	0,943	1,95E-03
*EHHADH*	ENSBTAG00000019625	enoyl-CoA, hydratase/3-hydroxyacyl CoA dehydrogenase	0,730	4,70E-02
*GOT1*	ENSBTAG00000011960	glutamic-oxaloacetic transaminase 1, soluble	0,547	3,19E-02
*GOT2*	ENSBTAG00000007172	glutamic-oxaloacetic transaminase 2, mitochondrial	0,747	6,27E-04
*HIBADH*	ENSBTAG00000001036	3-hydroxyisobutyrate dehydrogenase	0,427	7,16E-02
*HIBCH*	ENSBTAG00000007787	3-hydroxyisobutyryl-CoA hydrolase	0,492	6,46E-02
*HMGCL*	ENSBTAG00000021832	3-hydroxymethyl-3-methylglutaryl-CoA lyase	0,405	8,54E-02
*HPD*	ENSBTAG00000004175	4-hydroxyphenylpyruvate dioxygenase	1,532	3,56E-02
*HSD17B10*	ENSBTAG00000017779	hydroxysteroid (17-beta) dehydrogenase 10	0,734	2,39E-03
*IDO1*	ENSBTAG00000020602	indoleamine 2,3-dioxygenase 1	2,388	1,21E-02
*IVD*	ENSBTAG00000004409	isovaleryl-CoA dehydrogenase	0,574	3,25E-02
*KYNU*	ENSBTAG00000032277	Kynureninase	1,343	4,90E-02
*MDH1*	ENSBTAG00000019295	malate dehydrogenase 1, NAD (soluble)	0,818	1,36E-03
*NFS1*	ENSBTAG00000006962	NFS1 cysteine desulfurase	0,410	8.94E-02
*PIPOX*	ENSBTAG00000007946	pipecolic acid oxidase	1,219	2,16E-02
*SLC27A3*	ENSBTAG00000021862	solute carrier family 27 (fatty acid transporter), member 3	-1,150	4,79E-03

#### Carbohydrate metabolism

Differentially expressed genes pertaining to carbohydrate metabolism are shown in Table 8. Carbohydrates are energy-yielding nutrients, and several genes associated with carbohydrate degradation such as glycolytic reactions and mitochondrial transport and breakdown of pyruvate were up-regulated in the LPHE heifers. The fact that glycolysis-associated genes were DE between the two energy levels, suggests that the LPHE heifers had sufficient glucose available to direct some of it towards adipose tissue energy storage. This is not considered a common fate for glucose in ruminants which under most physiological conditions are dependent on *de novo* gluconeogenesis in the liver, as most of the glucose in the diet will be degraded by rumen microbes [32]. However, more recent studies have shown that hepatic *de novo* gluconeogenesis is highly prioritized in cows, and proceeds at a relatively high rate even in the absence of lactational demands or in the presence of available dietary glucose [35]. Thus, glucose directed towards adipose energy storage in HE heifers may originate from hepatic synthesis. The pentose-phosphate pathway is mainly associated with anabolic metabolism. It’s main functions are to provide ribose-5-phosphate for nucleic acid biosynthesis and reducing equivalents (NADPH) for the biosynthesis of fatty acids and steroids, as well as for the reduction of reactive oxygen species (ROS) [36]. The up-regulation of genes coding for pentose-phosphate pathway enzymes in HE heifers is in concordance with the up-regulation of genes associated with fatty acid synthesis and glutathione redox reactions (Tables 6 and 9, respectively). The up-regulation of genes associated with propionate, butyrate and ketone body metabolism and transport in LPHE heifers indicate an increased flux/transport of propionate and 3-hydroxybutyrate from blood into adipocytes. This is possible because the LPHE heifers had an increasingly higher energy intake per day than the LPLE heifers, and as a result microbial fermentation in ruminants causes the energy in feed to enter the animal’s bloodstream mainly as volatile fatty acids (VFA), not as glucose or fatty acids [37]. The difference in energy intake between HPHE and HPLE heifers was comparable to the LP energy contrast, however there was no significant up-regulation of genes pertaining to VFA transport between these two groups. A possible explanation is that the increased fraction of protein-rich feedstuffs in the experimental concentrate given to the HP groups, was substituted by barley, which is rich in starch, in the low-protein concentrate fed to the LP groups. This led to a difference in daily starch intake of approximately 130 grams between protein treatments. Starch is rapidly degraded into VFA (mainly propionate) in the rumen. The increased amount in the LP diets combined with the energy surplus in the HE roughage may have resulted in a sufficiently increased VFA flux from the rumen to the blood to evoke an up-regulation of 11 genes in LPHE heifers, enabling their adipose tissue to deal with the energy excess in the form of VFA and direct this energy towards fat deposition.

**Table 8:** Carbohydrate metabolism-associated genes differentially expressed between low-protein, high-energy- and low-protein, low-energy fed heifers.

Gene symbol	Ensembl Gene ID	Description	LogFC	FDR
*ALDOA*	ENSBTAG00000012927	aldolase A, fructose-bisphosphate	0,666	4,11E-03
*DLD*	ENSBTAG00000001908	dihydrolipoamide dehydrogenase	0,734	8,62E-03
*ENO1*	ENSBTAG00000013411	enolase 1, (alpha)	0,550	3,01E-02
*G6PD*	ENSBTAG00000019512	glucose-6-phosphate dehydrogenase	1,219	2,02E-05
*GPI*	ENSBTAG00000006396	glucose-6-phosphate isomerase	0,886	9,57E-04
*MCEE*	ENSBTAG00000035247	methylmalonyl CoA epimerase	0,939	8,90E-04
*MUT*	ENSBTAG00000014272	methylmalonyl CoA mutase	0,763	1,24E-02
*PCCB*	ENSBTAG00000015221	propionyl CoA carboxylase, beta polypeptide	0,741	8,80E-03
*PGAM1*	ENSBTAG00000012697	phosphoglycerate mutase 1 (brain)	0,604	9,47E-03
*PGD*	ENSBTAG00000013527	phosphogluconate dehydrogenase	1,038	2,52E-04
*PGK1*	ENSBTAG00000000894	phosphoglycerate kinase 1	0,623	9,08E-03
*TALDO1*	ENSBTAG00000010336	transaldolase 1	0,805	1,01E-03
*TKT*	ENSBTAG00000003758	Transketolase	0,770	5,11E-03
*TPI1*	ENSBTAG00000019782	triosephosphate isomerase 1	0,654	2,08E-02

**Table 9:** Antioxidant system-associated genes differentially expressed between low-protein, high-energy heifers and low-protein, low-energy heifers.

Gene symbol	Ensembl Gene ID	Description	LogFC	FDR
*ACTA1*	ENSBTAG00000046332	actin, alpha 1, skeletal muscle	-6,524	2,11E-03
*ACTG2*	ENSBTAG00000015441	actin, gamma 2, smooth muscle, enteric	-2,960	7,45E-03
*CAT*	ENSBTAG00000020980	Catalase	0,620	1,02E-02
*GCLC*	ENSBTAG00000015571	glutamate-cysteine ligase, catalytic subunit	0,778	1,94E-03
*GPX7*	ENSBTAG00000018281	glutathione peroxidase 7	-0,675	3,37E-02
*GSS*	ENSBTAG00000003504	glutathione synthetase	1,145	8,21E-04
*GSTM1*	ENSBTAG00000037673	glutathione S-transferase mu 1	1,020	2,27E-04
*Gstt1*	ENSBTAG00000040298	glutathione S-transferase, theta 1	1,308	1,81E-06
*Gstt3*	ENSBTAG00000008587	glutathione S-transferase, theta 3	1,151	8,65E-03
*MAF*	ENSBTAG00000044192	v-maf avian musculoaponeurotic fibrosarcoma oncogene homolog	-0,647	7,34E-02
*MAP2K1*	ENSBTAG00000033983	mitogen-activated protein kinase kinase 1	0,489	3,33E-02
*MGST1*	ENSBTAG00000008541	microsomal glutathione S-transferase 1	0,792	2,23E-02
*MGST3*	ENSBTAG00000010265	microsomal glutathione S-transferase 3	0,639	2,16E-02
*MRAS*	ENSBTAG00000001497	muscle RAS oncogene homolog	0,692	8,01E-03
*NQO1*	ENSBTAG00000020632	NAD(P)H dehydrogenase, quinone 1	1,302	2,25E-05
*PRDX1*	ENSBTAG00000003642	peroxiredoxin 1	0,777	3,77E-04
*PRDX6*	ENSBTAG00000004855	peroxiredoxin 6	0,542	1,47E-02
*PRKCZ*	ENSBTAG00000014119	protein kinase C, zeta	-1,589	1,44E-02
*SCARB1*	ENSBTAG00000014269	scavenger receptor class B, member 1	0,601	1,44E-02
*SOD1*	ENSBTAG00000018854	superoxide dismutase 1, soluble	0,490	8,54E-02
*SOD2*	ENSBTAG00000006523	superoxide dismutase 2, mitochondrial	0,673	2,39E-02
*SQSTM1*	ENSBTAG00000015591	sequestosome 1	0,724	2,34E-02
*UBE2K*	ENSBTAG00000020175	ubiquitin-conjugating enzyme E2K	0,452	9,97E-02
*USP14*	ENSBTAG00000019214	ubiquitin specific peptidase 14 (tRNA-guanine transglycosylase)	0,652	5,12E-02

#### Antioxidant functions

Differentially expressed genes pertaining to cellular antioxidant functions are shown in Table 9. Virtually any metabolic reaction involving oxygen may yield reactive oxygen species (ROS) as byproducts. Consequently, both mitochondria, endoplasmic reticulum (ER) and peroxisomes are major sites of ROS production in the cell [38]. In controlled quantities, these substances have a function in cell signaling and regulation [38]. However, accumulation of ROS will lead to a state of oxidative stress, causing uncontrolled oxidation of DNA and proteins in the cell and cellular dysfunction [39]. Therefore, it is imperative that cells have well-functioning systems to regulate and control the level of ROS at all times. The glutathione system plays an important role in the cellular defense against oxidative damage and harmful substances. Glutathione is synthetized by glutamate cysteine ligase (GCL, encoded by *GCLC*) and glutathione synthetase (GSS, encoded by *GSS*) from glycine and cysteine. It functions as an electron donor for reactive oxygen species (ROS), itself being dimerized to glutathione disulfide (GSSG) in the reaction. Glutathione disulfide may be recycled back to 2 GSH molecules by glutathione reductase, using NADPH as an H+ donor. In concordance with studies performed on mouse models, there was an up-regulation of *GCLC* and *GSS* and a down-regulation of the glutathione peroxidase gene *GPX7* in LPHE heifers [40, 41]. When examined in isolation, a down-regulation of *GPX7* indicates a decreased level of redox reactions producing GSSG and is associated with increases in oxidative stress [40], inflammatory status and insulin resistance [41]. However, as shown in Fig 6, a whole range of other antioxidant system genes were upregulated in LPHE heifers. Among these were several genes coding for glutathione-S-transferases (*GSTT1*, *GSTT3*, *GSTM1*, *MGST1* and *MGST3*). These enzymes represent another part of the glutathione system. They are multifunctional, and may act as peroxidases as well as catalyzing the conjugation of glutathione with unwanted substrates such as epoxides and lipid hydroperoxides, making them eligible for transport out of the cell [42]. The shift towards a conjugate-forming oxidative defense rather than one relying solely on redox reactions involving GSH/GSSG also indicates an increased cellular export of glutathione in HE heifers, as these conjugates are excreted and catabolized extracellularly [43]. An increased export of glutathione conjugates out of the cell may explain the need for increased production of GSH and the up-regulation of *GCLC* and *GSS*. An increased cellular excretion of harmful substances could also be expected from the upregulation of *EPHX2*. This gene codes for soluble epoxide hydrolase (SEH), a peroxisomal enzyme playing a role in cellular detoxification by converting lipid epoxides to trans-hydro-diols, which are eligible for conjugation and subsequent excretion [44].

The genes *SOD2* and *CAT* were also up-regulated in HE heifers. Superoxide dismutase (SOD) converts superoxides to H_2_O_2_, which may subsequently be reduced by either GPX or catalase. If regarded in isolation, the up-regulation of these two genes probably would represent a deterioration of the antioxidant defense in the HE heifers, as catalase exclusively binds H_2_O_2_, and at a low affinity, while *GPX* has the ability to reduce several other organic peroxides as well. For this reason, catalase has been regarded as a second line antioxidant defense, taking effect when peroxide levels become supraphysiological [45].

The combination of DEG representing mitochondrial energy metabolism pathways such as the TCA cycle and oxidative phosphorylation as well as the antioxidant system, is what makes the mitochondrial dysfunction pathway so highly ranked in the LPHE-LPLE contrast. During the last decade, increasing evidence has highlighted the associations between nutritional overload, mitochondrial changes in redox and energy state, insulin resistance and impaired adipocyte function [39–41, 45]. Although our HE heifers were clinically healthy, it is possible that these heifers, at 12 months of age and a BCS of almost 4 were on the verge of a harmful degree of fatness, and thus displayed a gene expression profile with similarities to that of morbidly obese individuals of other species. If this is the case, we may have revealed candidate genes for the early detection of obesity. Among these are genes coding for glutathione-S-transferases (*GSTT1*, *GSTT3*, *GSTM1*, *MGST1* and *MGST3*), *SOD2* and *CAT*.

### Expression differences between protein treatments

The differences between the protein treatments within each energy group were minimal. Only *CRYM*, coding for μ-crystallin, was DE for the HPLE-LPLE contrast, and was down-regulated in the HPLE heifers. The expression of *CRYM* is negatively associated with serum glucose levels, and positively with insulin sensitivity [46]. Therefore, it is interesting that this gene was DE in heifers fed different protein levels, but not different energy levels. Depending on passage rate and heat treatment, the ruminal digestibility of ground barley (present in varying amounts in our experimental concentrates) varies from 80–90% [47, 48], and the main product is propionate. Thus, only 10–20% of ingested starch may pass undegraded from the rumen to the intestine and can be absorbed into the blood as glucose. Given the higher starch intake of LPLE heifers, one would expect a higher blood glucose and a decreased *CRYM* expression in these animals, but it is possible that the association of both *CRYM* expression and blood glucose levels with insulin sensitivity may actually cause the opposite effect if insulin sensitivity is sufficiently high. If a difference in daily starch intake has indeed resulted in the observed up-regulation of *CRYM*, this indicates that certain parts of the metabolic regulation in heifers are sensitive to small changes in protein and carbohydrates available through feed, even on isocaloric diets, and that *CRYM* may be a sensitive indicator of glucose load in cattle. For the HPHE-LPHE contrast only *DSP* and *ACTA1* were DE, for which any notable association to dietary protein or starch are as of yet unknown. Thus, we have not found any genes to be differentially expressed as an effect of different feed protein levels regardless of energy level. This emphasizes further the dominant effect of total ration composition above the single effects of dietary energy or protein on the adipose tissue gene expression of growing heifers.

## Conclusions

Dietary energy content has a massive impact on the bovine adipose tissue transcriptome regulating several aspects of energy metabolism. The size of the effect is modulated by dietary protein content. The different protein levels applied in this study had only minor effects on adipose gene expression *per se*. Compared to heifers with a BCS of 3.63, heifers fed to a BCS of 3.95 at 12 mo of age displayed several transcriptional responses which have been associated with increased oxidative stress in other species. This study is a first step towards the identification of biomarkers for early-stage obesity (*GSTT1*, *GSTT3*, *GSTM1*, *MGST1*, *MGST3*, *SOD2*, *CAT*) and glucose load (*CRYM)* in cattle. However, certain biomarker identification will require further validation studies such as functional assays, lipidomics and proteomics. The current study underpins the importance of adipose transcriptional regulation of metabolism in growing cattle and highlights how total ration characteristics have a larger impact on adipose tissue transcriptomes than the single effects of protein and energy.

## Supporting information

S1 Table All differentially expressed genes from comparison of low-protein, high-energy- and low-protein, low-energy fed heifers. Pivot table of all differentially expressed genes found by comparing low-protein,high-energy (LPHE) and low-protein, low-energy (LPLE) fed heifers. Red rows show upregulated and green rows show downregulated genes in the adipose tissue of LPHE versus LPLE heifers.
(XLSX)

S2 Table All differentially expressed genes from comparison of low-protein, high-energy- and high-protein, low-energy fed heifers. Pivot table of all differentially expressed genes found by comparing high-protein,high-energy (HPHE) and high-protein, low-energy (HPLE) fed heifers. Red rows show upregulated and green rows show downregulated genes in the adipose tissue of HPHE versus HPLE heifers.
(XLSX)
